# Metabolic Exhaustion, p38 MAPK/Rac1 GTPase/MLKL Signaling, and Ca^2+^ Influx Mediate Solasodine-Triggered Eryptosis

**DOI:** 10.33549/physiolres.935854

**Published:** 2026-08-01

**Authors:** Iman A. ALAJEYAN, Jawaher ALSUGHAYYIR, Mohammad A. ALFHILI

**Affiliations:** 1 Department of Clinical Laboratory Sciences, College of Applied Medical Sciences, King Saud University, Riyadh, Saudi Arabia

**Keywords:** Solasodine, Eryptosis, p38 MAPK, Rac1 GTPase, MLKL

## Abstract

Solasodine (SOL) is a steroidal glycoalkaloid that shows a wide range of biological effects, most notably anticancer activities. Eryptosis is the programmed cell death of erythrocytes that leads to anemia. Specifically, chemotherapy-induced anemia can be precipitated in part by eryptosis triggered by anticancer agents. We hypothesize that the cytotoxic effects of SOL observed in cancer cells are nonspecific and extend to human erythrocytes. Eryptosis following SOL exposure was detected by fluorescence-assorted cell sorting analysis in erythrocytes from healthy volunteers. Phosphatidylserine (PS) translocation and cellular volume were measured using annexin-V-FITC and forward scatter (FSC), respectively. Fluo-4/AM was used to detect cytoplasmic Ca^2+^ and H_2_DCFDA was employed to probe oxidative stress. Hemolysis was also assessed by hemoglobin leakage. In addition, different incubation media and inhibitors were tested for their potential influence on SOL activity. Exposure to SOL led to modest but significant hemolysis and was paralleled by significant PS translocation, elevated FSC, and echinocyte morphology. Energy restitution through ATP, guanosine, and adenine significantly reversed PS translocation as did the removal of extracellular Ca^2+^ and the dissipation of the cell membrane K^+^ gradient. Co-treatment of erythrocytes with SOL and SB203580, NSC 23766, necrosulfonamide, caffeine, and melatonin significantly inhibited SOL-induced PS translocation. In conclusion, SOL is a novel pro-eryptotic compound whose activity is mediated through energy exhaustion, Ca^2+^ influx, and cytosolic KCl depletion, and requires p38 MAPK/Rac1 GTPase/MLKL signaling. Metabolic substrates, cation channel modulators, and targeted inhibition provide protective adjuncts to improve the therapeutic index of SOL as it advances toward translational applications.

## Introduction

Steroidal glycoalkaloids are secondary plant metabolites in various foods, including tomatoes, potatoes, and eggplants. Glycoalkaloids and their hydrolysis products, aglycons, have numerous biological effects despite their toxic character [1]. The two most significant glycoalkaloids found in solanaceous plants are solasonine and solamargine, whose main aglycone is solasodine (SOL). This spiroketal alkaloid sapogenin contains a structural component of a C27 cholestane skeleton where a 1–5 carbohydrate side chain is attached at the aglycone's 3-OH region [2]. SOL has been shown to have antifungal [3], anticonvulsant [4], anti-inflammatory [5], and antimicrobial [6] effects.

Importantly, the impact of SOL on cancer cells has been extensively examined in previous research. Significant inhibitory effects on cell growth have been reported for SOL in nasopharyngeal [7], breast [8], oral [9], gastric [10], colorectal [11], liver [12], ovarian [13], and lung [14] cancer cells. Specifically, the anticancer activity of SOL depends on suppressing AKT/GSK-3b/β-catenin, Hedgehog/Gli1, and NF-κB-p65 signaling, reducing mitochondrial membrane potential, and stimulation of cell death through apoptosis, autophagy-dependent cell death, and ferroptosis.

Although red blood cells (RBCs) lack intracellular organelles, they can still execute programmed cell death in the form of eryptosis and necroptosis. Eryptosis is a physiological process through which senescent or dysfunctional erythrocytes are labeled for recognition by phagocytes. This labeling is achieved through the breakdown of the asymmetrical distribution of phospholipids during which phosphatidylserine (PS) translocates to the outer membrane leaflet. Phagocytes then bind externalized PS through specific receptors to engulf dead RBCs and minimize the risk of hemolysis and anemia. Eryptosis may also be pathological if it prematurely occurs at a faster rate than the compensatory erythropoietic response. This can be seen in a plethora of conditions such as kidney disease, sepsis, cancer, diabetes, and cardiovascular disease. Additionally, eryptosis is triggered by a growing number of medications, pesticides, toxins, and other xenobiotics [15].

Anemia is common among cancer patients and arises due to myelosuppressive chemotherapy [16] and excessive eryptosis [17]. This work tested the hypothesis that SOL adversely affects non-target tissue, specifically erythrocytes, due to nonspecific toxicity. The aim of the current study was to evaluate the erythrotoxic biochemical mechanisms of SOL in erythrocytes which constitutes a clinically relevant toxicity model [18].

## Materials and Methods

### Erythrocytes, chemicals, and solutions

The protocol for this work conforms to the guidelines published by the Eryptosis Study Consortium [15], and has been approved by the Ethics Committee of King Saud University (E-23-7552). In accordance with the Declaration of Helsinki, blood samples anticoagulated with EDTA or lithium heparin were taken from 23 healthy participants with normal complete blood count (CBC) and body mass index (BMI) after giving verbal and written informed consent. The donors were 15 males and 8 females, with an age range of 25 to 38 years. RBCs were isolated by centrifugation of whole blood at 3,000 RPM for 20 min at room temperature and resuspended in Ca^2+^-free Ringer solution. All chemicals, kits, and reagents were of the highest purity and were procured from Solarbio Life Science (Beijing, China). SOL (CAS #126-17-0) was prepared as a 40 mM (16.0 mg/ml) stock solution in ethanol and stored as aliquots at −80 °C. Aliquots were only used once to avoid repeated freezing and thawing. SOL exposure (40–500 μM) was conducted in Ringer solution with a hematocrit of 5 % for 48 h at 37 °C. This range was chosen based on previously published studies investigating the anticancer effects of SOL which used concentrations from 10 to 240 μM [8, 12]. Since SOL largely remains at the preclinical stage, circulating plasma levels are not yet available. Therefore, the concentrations used herein are primarily intended for toxicological assessment rather than direct physiological plasma equivalents. Erythrocytes incubated with deionized water and 1 % ethanol were used as a positive and negative control, respectively.

### Eryptosis

All eryptotic markers were measured in 10,000 cells using fluorogenic probes excited by a blue light of 488 nm to emit a green light of 520 nm. Northern Lights flow cytometer (Cytek Biosciences, Fremont, CA, USA) was used for all assays. PS translocation was captured by labeling membranes with annexin-V-FITC whereas cytoplasmic Ca^2+^ and ROS were measured by Fluo4/AM and 2',7'-dichlorodihydrofluorescein diacetate (H_2_DCFDA), respectively. In the annexin assay, 50 μl of cells were added to 150 μl of Ringer buffer containing 5 mM CaCl_2_ and 1 % annexin-V-FITC and incubated at room temperature in the dark for 10 min. In the Ca^2+^ assay, 50 μl of cells were added to 150 μl of Ringer buffer containing 5 mM CaCl_2_ and 2 μM Fluo4/AM incubated at 37°C in the dark for 30 min. In the oxidative stress assay, 50 μl of cells were added to 150 μl of standard Ringer buffer containing 10 μM H_2_DCFDA and incubated at 37 °C in the dark for 30 min. After incubation, cells were washed twice in PBS (13,000 *x g*, 1 min) to remove unbound Fluo4 and DCF before analysis in a final volume of 300 μl of PBS. Forward scatter (FSC) was also measured as a marker of cell size [19]. Since RBCs are relatively homogenous, lack nuclei, and occupy a narrow FSC/SSC region, cells were identified by acquisition thresholds and no post-acquisition gating was required. Quantification for eryptotic cells was expressed as a percentage of annexin V-positive cells identified by an analysis marker placed according to control cells. FSC, Fluo4, and DCF signals were expressed as mean fluorescence intensity (MFI) values in arbitrary units.

### Hemolysis

Extracellular hemoglobin was photometrically measured at 405 nm by LMPR-A14 microplate reader (Labtron, Surrey, UK) [20]. Cells were centrifuged at 13,000 RPM for 1 min at room temperature and 200 μl of the supernatant were added to a 96-well plate to measure hemoglobin content.

### Scanning electron microscopy (SEM)

Fixed cells in 2 % glutraldehyde were stained with 1 % osmium tetraoxide, serially dried in 50–100 % ethanol, gold-coated, and finally observed under JSM-7610F scanning electron microscope operating at 15.0 kV (JEOL, Tokyo, Japan) [21].

### Functional assays

Cells were exposed to 100 or 200 μM SOL in the presence or absence of 100 μM SB203580 (p38 MAPK inhibitor), 20 μM of D4476 (CK1α inhibitor), 0.5 μM of necrosulfonamide (NSA; MLKL inhibitor), 5 μM of melatonin, 1 μM of staurosporine (PKC inhibitor), 20 μM of BAPTA-AM (cell-permeable Ca^2+^ chelator), 100 μM of NSC23766 (Rac1 GTPase inhibitor), 500 μM of ATP, 2 mM guanosine, or 2 mM adenine [15].

### Statistical analysis

Data are displayed as means ± SD of three independent experiments (*n* = 9). Statistical analysis was carried out by Prism 9.0 (GraphPad, San Diego, CA, USA) using one-way ANOVA with Dunnett’s or Tukey’s test. Significance was set at a *p*-value of less than 0.05.

## Results

### SOL stimulates eryptosis with cell swelling and membrane blebbing

**Fig. 1:**
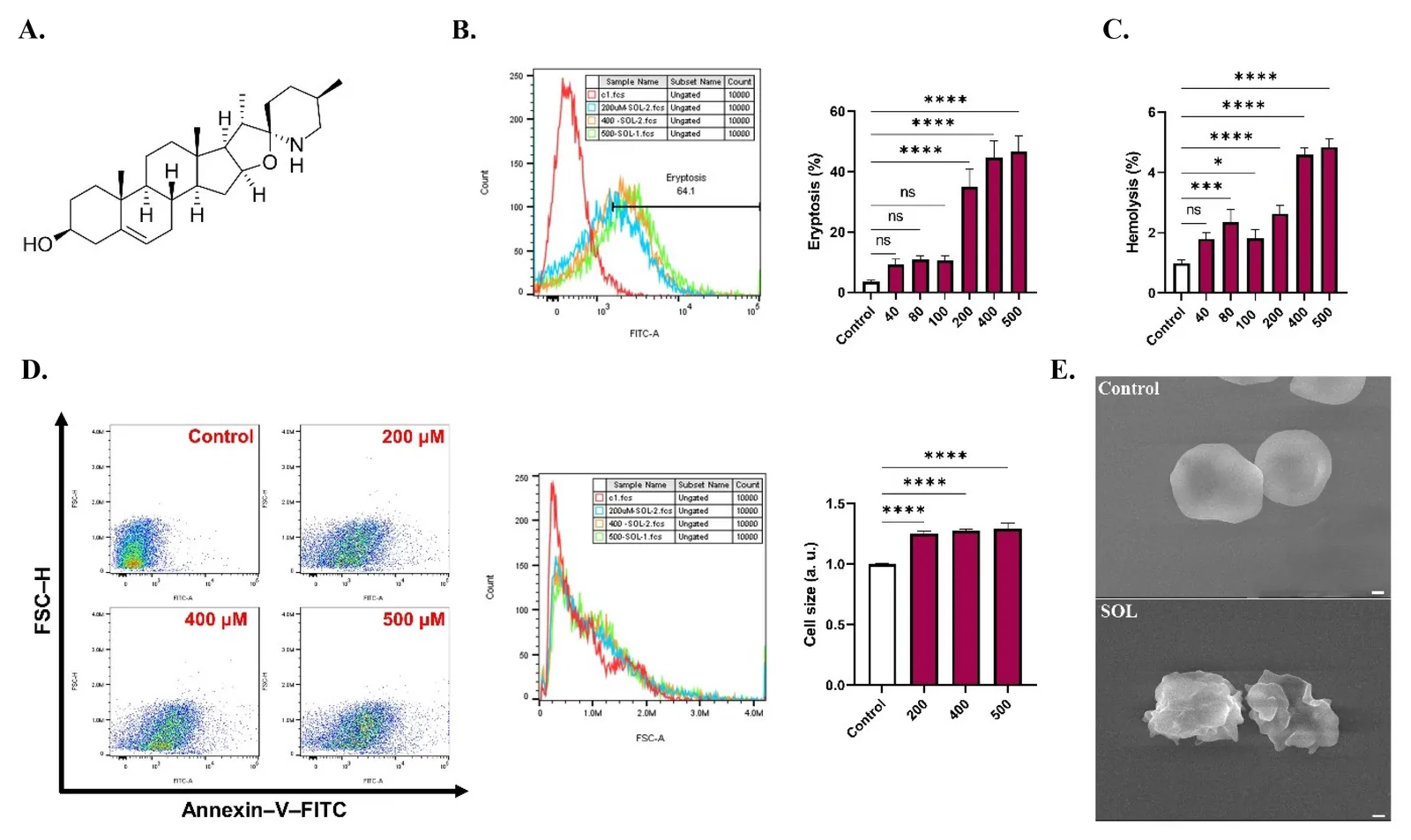
Effect of SOL on eryptosis. (**A**) Chemical structure of SOL. (**B**) Representative annexin-V-FITC histograms and arithmetic means of eryptotic cells. (**C**) Arithmetic means of the percentage of haemolytic cells. Results are shown as means ± SD (*n* = 9). (**D**) Representative dot plots (FSC-H *vs*. annexin-V-FITC), overlayed FSC-A histograms, and arithmetic means of cell size. (**E**) Representative electron microscopy micrographs (X5,000; Scale bar: 1 μm) of control and treated (100 μM) cells. ns indicates no statistical significance, while ^*^(*p* <0.05), ^***^(*p* <0.001), and ^****^(*p* <0.0001).

The primary aim of this study was to assess the toxicity of SOL (Fig. 1A) in RBCs. Fig. 1B demonstrates that treatment with SOL at 200, 400, and 500 μM showed a significant increase (*p* <0.0001) in the percentage of annexin-V-binding erythrocytes. Modest but significant hemolytic activity was also observed (Fig. 1C). Eryptosis is characterized by cell shrinkage, hence FSC was assessed. Interestingly, cells exposed to 200, 400, and 500 μM SOL exhibited rather significant cell swelling (*p* <0.0001) as seen in Fig. 1D. Another morphological feature of eryptosis is the formation of membrane blebs. Accordingly, exposure to 100 μM SOL resulted in echinocyte transformation as revealed by electron microscopy (Fig. 1E).

### SOL-induced eryptosis is prevented by energy restitution

**Fig. 2:**
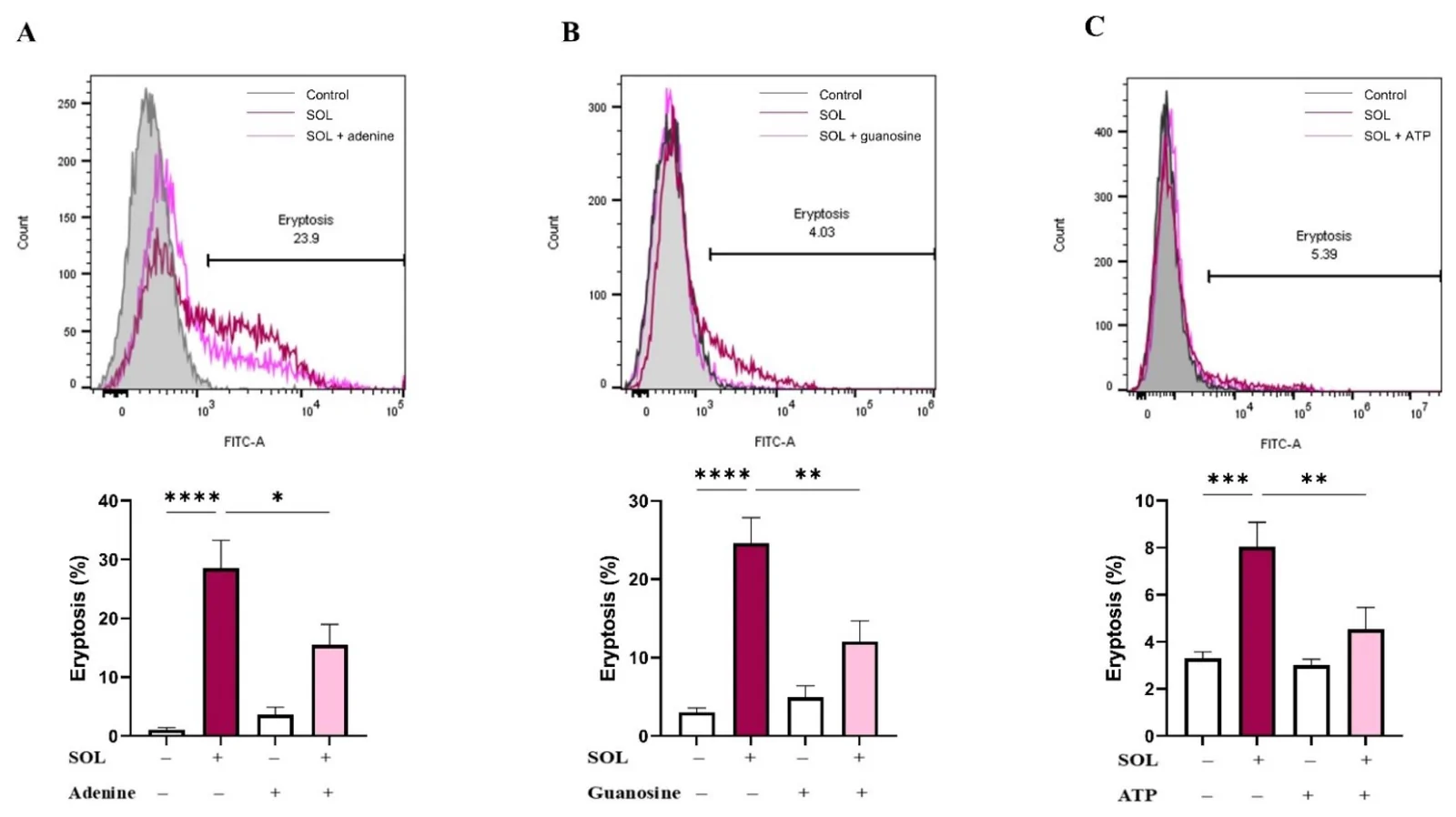
Ameliorative effect of energy restitution on SOL-induced eryptosis. Representative annexin-V-FITC histograms and arithmetic means of eryptotic cells in the absence and presence of (**A**) adenine (2 mM), (**B**) guanosine (2 mM), and (**C**) ATP (0.5 mM). Results are shown as means ± SD (*n* = 9). ns indicates no statistical significance, while ^*^(*p* <0.05), ^**^(*p* <0.01), ^***^(*p* <0.001), and ^****^(*p* <0.0001).

Eryptosis is also triggered by energy depletion, so a subsequent set of experiments explored the potential protective effect of metabolic substrates against SOL toxicity. We found that the addition of adenine (Fig. 2A), guanosine (Fig. 2B), and ATP (Fig. 2C) significantly inhibited PS translocation.

### SOL-induced eryptosis is sensitive to p38 MAPK and MLKL inhibition

**Fig. 3:**
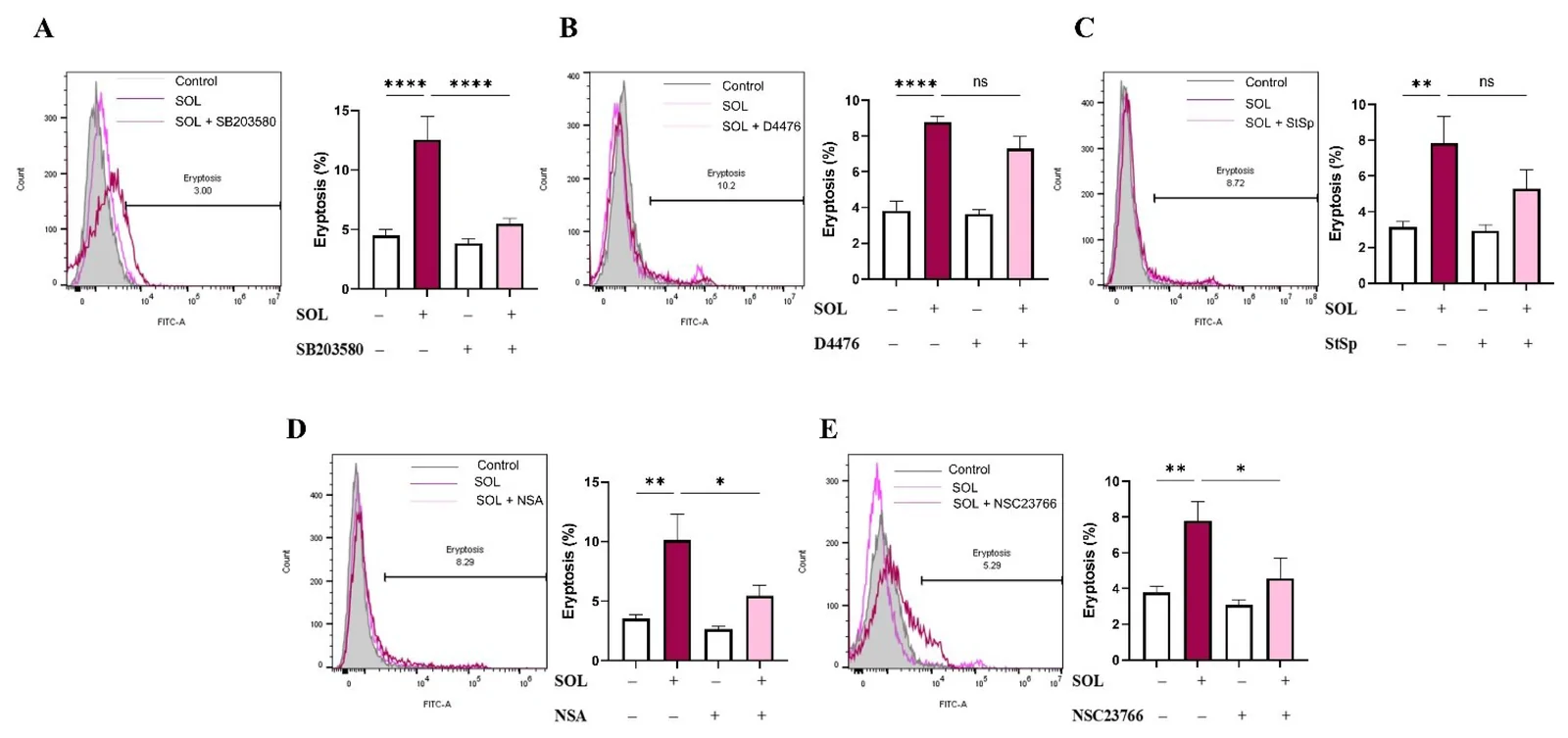
Signaling enzymes in SOL-induced eryptosis. Representative annexin-V-FITC histograms and arithmetic means of eryptotic cells in the absence and presence of (**A**) SB203580 (p38 MAPK inhibitor; 100 μM), (**B**) D4476 (CK1α inhibitor; 20 μM), (**C**) staurosporin (StSp, PKC inhibitor; 1 μM), (**D**) necrosulfonamide (NSA, MLKL inhibitor; 0.5 μM), and (E) NSC 23766 (Rac1 inhibitor; 100 μM). Results are shown as means ± SD (*n* = 9). ns indicates no statistical significance, while ^*^(*p* <0.05), ^**^(*p* <0.01), and ^****^(*p* <0.0001).

Next, we probed signaling enzymes involved in SOL-induced eryptosis. Using SB203580 to inhibit p38 MAPK (Fig. 3A) significantly reversed PS exposure (*p* <0.0001) unlike D4476 (Fig. 3B) and staurosporine (Fig. 3C) which inhibit CK1α and PKC, respectively. Likewise, significant protection (*p* <0.05) was also seen in the presence of MLKL inhibitor, necrosulfonamide (Fig. 3D) and Rac1 inhibitor NSC23766 (Fig. 3E).

### SOL-induced eryptosis is ameliorated by caffeine and melatonin

**Fig. 4:**
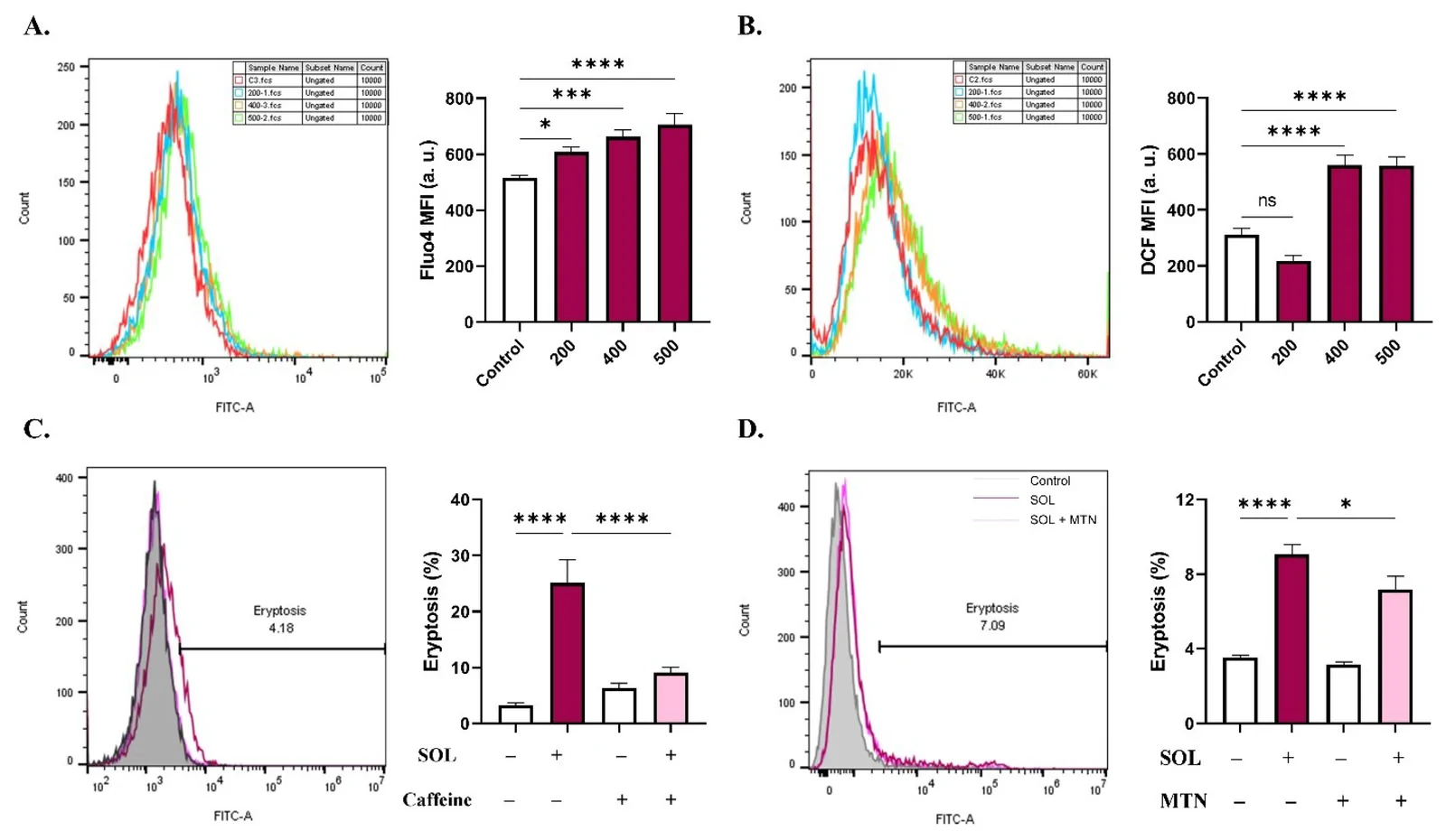
Caffeine and melatonin abrogate SOL-induced eryptosis. (**A**) Representative Fluo4 histograms and arithmetic means of Fluo4 fluorescence intensity (MFI). (**B**) Representative DCF histograms and arithmetic means of DCF-positive cells. (**C**) Representative annexin-V-FITC histograms and arithmetic means of eryptotic cells in the absence and presence of caffeine (0.5 mM). (**D**) Representative annexin-V-FITC histograms and arithmetic means of eryptotic cells in the absence and presence of melatonin (MTN; 5 μM). Results are shown as means ± SD (n = 9). ^*^(*p* <0.05), ^***^(*p* <0.001), and ^****^(*p* <0.0001).

Ca^2+^ buildup and oxidative stress are recognized as major mechanisms underlying eryptosis, and both were involved in SOL-induced PS translocation, as can be seen in Fig. 4A (significantly increased Fluo4 fluorescence) and 4B (significantly increased DCF-positive cells), respectively. Additionally, the Ca^2+^ channel modulator, caffeine (*p* <0.0001; Fig. 4C), and the antioxidant, melatonin (*p* <0.05; Fig. 4D) alleviated the pro-eryptotic activity of SOL.

### Extracellular Ca^2+^ exclusion and membrane depolarization reverse SOL-induced eryptosis

**Fig. 5:**
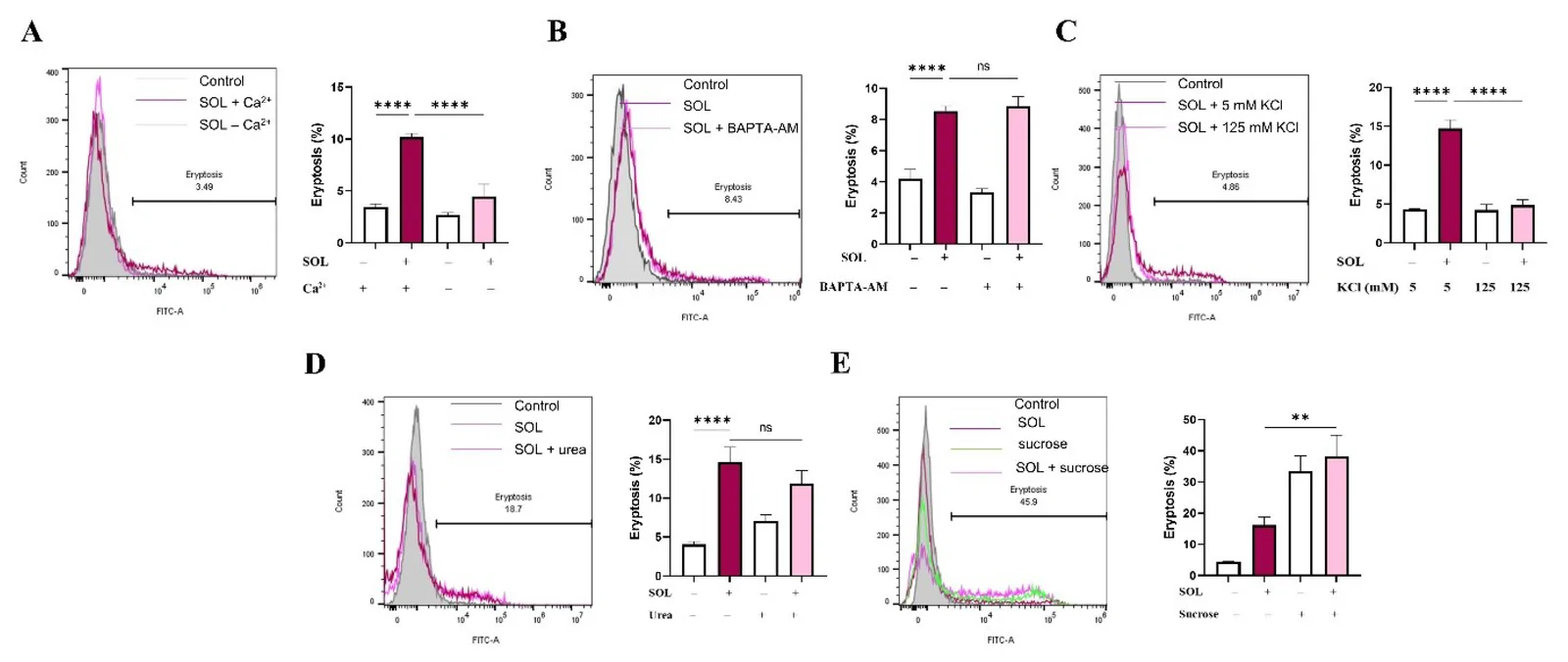
SOL-induced eryptosis requires Ca^2+^ entry and membrane hyperpolarization. Representative annexin-V-FITC histograms and arithmetic means of eryptotic cells in the absence and presence of (**A**) extracellular Ca^2+^, (**B**) BAPTA-AM (20 μM), (**C**) 5 or 125 mM KCl, (**D**) urea (300 mM), and (**E**) sucrose (250 mM). Results are shown as means ± SD (*n* = 9). ns indicates no statistical significance while ^**^(*p* ≤0.01) and ^****^(*p* <0.0001).

Loss of K^+^ upon Ca^2+^ accumulation is a permissive signal for eryptosis. Thus, we were prompted to examine the participation of Ca^2+^ entry and KCl efflux in SOL-induced eryptosis. The results indicate that suspension of cells in Ca^2+^-free Ringer buffer (Fig. 5A) but not co-incubation with BAPTA-AM (Fig. 5B), significantly reduced SOL toxicity (*p* <0.0001). In addition, KCl-rich Ringer buffer (Fig. 5C) significantly inhibited PS translocation induced by SOL (*p* <0.0001). Conversely, while the addition of urea had no effect (Fig. 5D), sucrose rather exacerbated SOL-induced PS translocation (*p* <0.01; Fig. 5E).

**Fig. 6:**
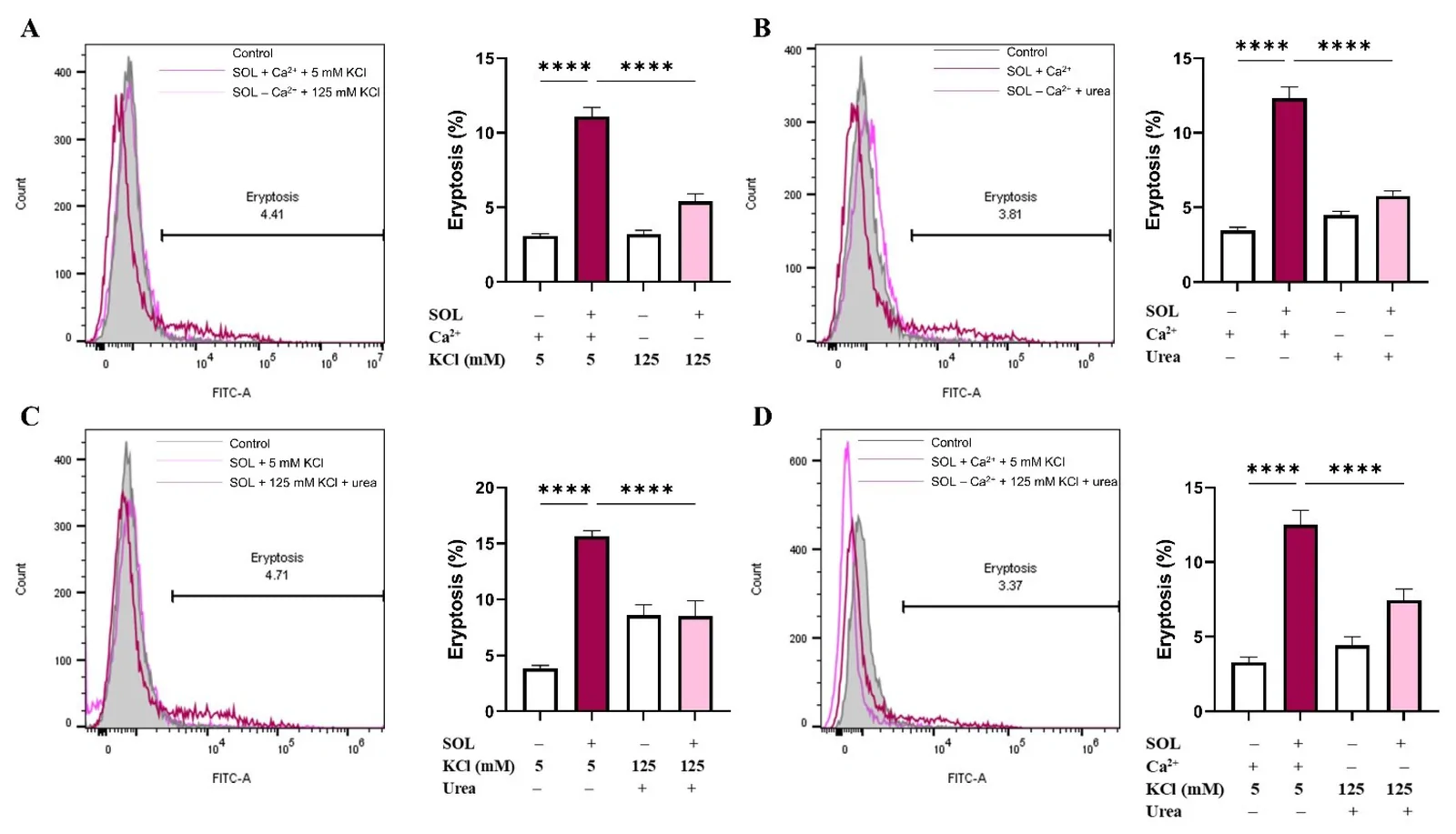
Effect of combined modifications of Ringer buffer on SOL-induced eryptosis. Representative annexin-V-FITC histograms and arithmetic means of eryptotic cells in (**A**) the presence and absence of extracellular Ca^2+^ with 5 or 125 mM KCl, (**B**) the presence and absence of extracellular Ca^2+^ with or without urea (300 mM), (**C**) 5 or 125 mM KCl with or without urea, and (**D**) in the presence and absence of extracellular Ca^2+^, with 5 or 125 mM KCl, and with and without urea. Results are shown as means ± SD (*n* = 9). ^****^(*p* <0.0001).

Based on these results, further experiments were conducted to investigate the impact of combined modifications to the incubation media on SOL cytotoxicity. It was noted that the eryptotic activity of SOL was abrogated (*p* <0.0001) upon simultaneous Ca^2+^ exclusion and membrane depolarization (Fig. 6A), Ca^2+^ exclusion in the presence of urea (Fig. 6B), membrane depolarization and urea addition (Fig. 6C), and concurrent Ca^2+^ exclusion, membrane depolarization, and addition of urea (Fig. 6D).

## Discussion

Eryptosis is increasingly being recognized as a key pathological process of many conditions including anemia. This study is the first to provide experimental evidence that SOL triggers eryptosis which indicates that the cytotoxicity of SOL is nonspecific to cancer cells. This finding is seminal for the clinical prospects of SOL since chemotherapy-related anemia is among the most serious adverse drug reactions [22]. In particular, adjunctive cytoprotective strategies may improve the safety profile of SOL although *in vivo* pharmacokinetic studies remain necessary to assess whether the concentrations used and the toxic phenotypes observed are clinically achievable upon systemic exposure.

Similar to other xenobiotics, this work demonstrates that SOL has weak but significant hemolytic action in human erythrocytes (Fig. 1). Naked heme has recently been shown to be thrombogenic by stimulating ferroptosis and NETosis to elicit platelet activation [23]. Nonetheless, Jayakumar *et al*. [24] reported that SOL showed antihemolytic activity against H_2_O_2_. This dual effect has been reported for picolinic acid [25], which was anti-hemolytic against osmotic stress but hemolytic in isotonic conditions.

Following SOL treatment, PS translocation to the erythrocyte surface was observed (Fig. 1) indicating cell membrane scrambling parallel to apoptosis in nucleated cells [1–3]. Since the erythrocytes used in this investigation were collected from healthy individuals, it is possible that clinical conditions involving accelerated eryptosis could increase sensitivity to SOL. The exposure of PS on the surface of eryptotic cells may have two main pathophysiological consequences. First, it fosters the binding of eryptotic cells to specific receptors on macrophages leading to their degradation. Second, it enhances their adherence to the vascular wall, which could obstruct the microcirculation [15].

Functional assays revealed that restoring energy protects cells from SOL toxicity (Fig. 2), which aligns with established evidence of the pivotal role of glycolytic integrity in erythrocyte survival. Thus, depleting intracellular energy reservoirs may play a significant role in SOL-induced eryptosis. Erythrocytes require a continuous supply of energy through glycolysis, and their deformability is adversely affected when their energy resources are depleted [26,27]. Also, excessive Ca^2+^ pump activity results in fast ATP consumption, attributed to Ca^2+^ influx [28] (Fig. 5). Importantly, guanosine and adenine act as precursors to GTP and ATP, respectively [29,30]. Guanosine, in particular, has cytoprotective functions attributed to its antioxidant effects [31], similar to melatonin (Fig. 4).

Our results indicate that SB203580, NSC23766, and NSA each independently reversed the pro-eryptotic effect of SOL (Fig. 3). Signaling cascades through these enzymes modulate a host of downstream effectors. Osmotic stress activates p38 MAPK and Ca^2+^ influx [32], shown to be a required step in SOL activity (Fig. 5). Rac1 regulates the cytoskeleton [15] and may therefore mediate membrane blebbing (Fig. 1), although a crosstalk with Ca^2+^ cannot be excluded [33]. Further, the participation of MLKL implicates necroptosis in SOL-induced RBC death although it can also signal for apoptosis [34]. Alarmingly, necroptosis is triggered by several xenobiotics, bacterial toxins, *in vitro* storage, and hyperglycemic stress; and has been implicated in stroke, tumor, inflammation, and infection [35].

PS externalization was ameliorated by caffeine (Fig. 4), which is among the few compounds known to exert anti-eryptotic effects by inhibiting Ca^2+^ influx [36]. In congruence, eryptosis was selectively alleviated in cells deprived of extracellular Ca^2+^ but in those co-treated with BAPTA-AM (Fig. 5). Taken together, these results identify Ca^2+^ entry, as opposed to downstream Ca^2+^ effects, as a required step in SOL-mediated eryptosis. Upon Ca^2+^ entry, Ca^2+^-dependent phospholipid scramblase activity becomes dysregulated leading to characteristic membrane scrambling. Moreover, Ca^2+^-sensitive K^+^ channels are activated which drive K^+^, Cl^–^, and water out of the cell following membrane hyperpolarization. Of note, our results show that stabilizing membrane potential by dissipating this K^+^ gradient significantly rescues cells from PS translocation (Fig. 5). Interestingly, our results indicate that erythrocytes exposed to SOL showed increased cell volume (Fig. 1), which seems to suggest that its primary insult is osmotic.

While the absence of appreciable shrinkage might seem contradictory to the protection offered by reducing the transmembrane K^+^ gradient (Fig. 5), we offer two plausible interpretations of this finding. First, water efflux might have been accompanied by sufficient Na^+^ entry, thereby minimizing net volume loss [37]. Second, cells undergoing osmotic swelling may attempt to restore their physiological size by opening K^+^ and Cl^−^ channels to export ions [38] which is a permissive step for downstream eryptotic signaling. Collectively, these observations suggest that PS translocation and net volume changes are not invariably coupled under all ionic stress conditions as previously reported [39, 40]. Altogether, these mechanistic insights argue for the exploration of selective Ca^2+^ and K^+^ channel blockers as therapeutic adjuvants for SOL.

Another inhibitor of SOL-induced eryptosis is melatonin (Fig. 4), which has antioxidant [41] and anti-inflammatory properties. The protection conferred by melatonin may be linked to suppressing prostaglandin E_2_ which activates Ca^2+^ channels to promote PS exposure [42]. This, along with other mechanisms, may underlie the appearance of echinocytes and membrane blebs in SOL-treated cells (Fig. 1). Specifically, cytoskeletal weakening may arise from Ca^2+^ entry (Fig. 5), ATP depletion (Fig. 2), kinase stimulation (Fig. 3), calpain activation [43], osmotic stress and swelling (Fig. 1), and others. This result is in agreement with that reported by Manrique-Moreno *et al*. [44], who observed echinocytes following SOL treatment. Blebbing consumes membrane reserve, reducing the surface-area-to-volume ratio which significantly limits deformability. Membrane elasticity is also diminished, with higher cellular fragility, and an increased risk of occlusive lesions [15].

SOL toxicity was significantly enhanced when sucrose was added to the incubation medium (Fig. 5), revealing an additive effect. Congruently, sucrose stimulates the breakdown of the PS asymmetry in erythrocytes through the formation of ceramide and Ca^2+^ signaling. Our investigations also indicate that urea on its own was unable to confer protection against SOL, possibly ruling out the requirement of sphingomyelinase or several transport systems, most notably Na^+^/K^+^/2Cl^−^ cotransport [45].

## Conclusions

In conclusion, this study demonstrates that SOL triggers eryptosis characterized by cell swelling, membrane scrambling and blebbing, metabolic exhaustion, Ca^2+^ entry, oxidative stress, and signaling cascades involving p38, Rac1, and MLKL. Therapeutic strategies to mitigate SOL-mediated off-target erythrocyte injury include cation transport modulation, antioxidants, energy substrates, and enzyme inhibitors. Confirmation of the erythrotoxicity of SOL in animal models could further establish the clinical relevance of these findings and inform the development and validation of safer therapeutic applications.

## References

[1] Smith SW, Giesbrecht E, Thompson M, Nelson LS, Hoffman RS (2008). Solanaceous steroidal glycoalkaloids and poisoning by Solanum torvum, the normally edible susumber berry. Toxicon.

[2] Jayakumae KMK (2015). Solanum Alkaloids and their Pharmaceutical Roles: A Review. J Anal Pharm Res.

[3] Kusano G, Takahashi A, Sugiyama K, Nozoe S (1987). Antifungal properties of solanum alkaloids.

[4] Chauhan K, Sheth N, Ranpariya V, Parmar S (2011). Anticonvulsant activity of solasodine isolated from Solanum sisymbriifolium fruits in rodents. Pharm Biol.

[5] Pandurangan A, Khosa RL, Hemalatha S (2011). Anti-inflammatory activity of an alkaloid from Solanum trilobatum on acute and chronic inflammation models. Nat Prod Res.

[6] Kumar P, Sharma B, Bakshi N (2009). Biological activity of alkaloids from Solanum dulcamara L. Nat Prod Res.

[7] Wang J, Wang D, Ma S, He B, Lu J, Guo Z (2025). Solasodine suppresses nasopharyngeal carcinoma progression by inducing ferroptosis. Sci Rep.

[8] Chen J, Ma D, Zeng C (2022). Solasodine suppress MCF7 breast cancer stem-like cells via targeting Hedgehog/Gli1. Phytomedicine.

[9] Bharathiraja P, Balamurugan K, Govindasamy C, Prasad NR, Pore PM (2024). Solasodine targets NF-κB signaling to overcome P-glycoprotein mediated multidrug resistance in cancer. Exp Cell Res.

[10] Su K, Yao X, Guo C (2023). Solasodine suppresses the metastasis of gastric cancer through claudin-2 via the AMPK/STAT3/NF-κB pathway. Chem Biol Interact.

[11] Zhuang YW, Wu CE, Zhou JY (2017). Solasodine inhibits human colorectal cancer cells through suppression of the AKT/glycogen synthase kinase-3β/β-catenin pathway. Cancer Sci.

[12] Lee KR, Kozukue N, Han JS (2004). Glycoalkaloids and metabolites inhibit the growth of human colon (HT29) and liver (HepG2) cancer cells. J Agric Food Chem.

[13] Xu XH, Zhang LL, Wu GS (2017). Solasodine Induces apoptosis, affects autophagy, and attenuates metastasis in ovarian cancer cells. Planta Med.

[14] Shen KH, Hung JH, Chang CW, Weng YT, Wu MJ, Chen PS (2017). Solasodine inhibits invasion of human lung cancer cell through downregulation of miR-21 and MMPs expression. Chem Biol Interact.

[15] Tkachenko A, Alfhili MA, Alsughayyir J (2025). Current understanding of eryptosis: mechanisms, physiological functions, role in disease, pharmacological applications, and nomenclature recommendations. Cell Death Dis.

[16] Tas F, Eralp Y, Basaran M (2002). Anemia in oncology practice: relation to diseases and their therapies. Am J Clin Oncol.

[17] Lang E, Bissinger R, Qadri SM, Lang F (2017). Suicidal death of erythrocytes in cancer and its chemotherapy: A potential target in the treatment of tumor-associated anemia. Int J Cancer.

[18] Tkachenko A (2024). Hemocompatibility studies in nanotoxicology: Hemolysis or eryptosis? (A review). Toxicol In Vitro.

[19] Jemaà M, Fezai M, Bissinger R, Lang F (2017). Methods Employed in Cytofluorometric Assessment of Eryptosis, the Suicidal Erythrocyte Death. Cell Physiol Biochem.

[20] Hopp MT, Vaidya SM, Grimmig KM (2024). Quantitative analysis of heme and hemoglobin for the detection of intravascular hemolysis. Anal Chim Acta.

[21] Carrera-Bravo C, Zhou T, Chu T (2025). Chloroquine induces eryptosis in P. falciparum-infected red blood cells and the release of extracellular vesicles with a unique protein profile. Front Cell Infect Microbiol.

[22] Bissinger R, Schumacher C, Qadri SM (2016). Enhanced eryptosis contributes to anemia in lung cancer patients. Oncotarget.

[23] NaveenKumar SK, Hemshekhar M, Sharathbabu BN, Kemparaju K, Mugesh G, Girish KS (2023). Platelet activation and ferroptosis mediated NETosis drives heme induced pulmonary thrombosis. Biochim Biophys Acta Mol Basis Dis.

[24] Jayakumar KSSS, Murugan K (2016). Evaluation of mitigating effect of purified solasodine from Solanum mauritianum against hydrogen peroxide induced oxidative damage in human erythrocytes. International Journal of Applied Biology and Pharmaceutical Technology.

[25] Alghareeb SA, Alsughayyir J, Alfhili MA (2025). Picolinic acid, a tryptophan metabolite, triggers cellular senescence by targeting NOS/p38 MAPK/CK1α/MLKL signaling and metabolic exhaustion in red blood cells. Toxicol Res.

[26] Gov NS, Safran SA (2005). Red blood cell membrane fluctuations and shape controlled by ATP-induced cytoskeletal defects. Biophys J.

[27] Brun JF, Varlet-Marie E, Myzia J, Raynaud de Mauverger E, Pretorius E (2021). Metabolic Influences Modulating Erythrocyte Deformability and Eryptosis. Metabolites.

[28] Bogdanova A, Makhro A, Wang J, Lipp P, Kaestner L (2013). Calcium in red blood cells-a perilous balance. Int J Mol Sci.

[29] Dunn Jacob, Grider Michael H Physiology, Adenosine Triphosphate. StatPearls.

[30] Hesketh A, Oliver SG (2019). High-energy guanine nucleotides as a signal capable of linking growth to cellular energy status via the control of gene transcription. Curr Genet.

[31] Quincozes-Santos A, Bobermin LD, Souza DG, Bellaver B, Gonçalves CA, Souza DO (2014). Guanosine protects C6 astroglial cells against azide-induced oxidative damage: a putative role of heme oxygenase 1. J Neurochem.

[32] Gatidis S, Zelenak C, Fajol A (2011). p38 MAPK activation and function following osmotic shock of erythrocytes. Cell Physiol Biochem.

[33] Baker MJ, Abba MC, Garcia-Mata R, Kazanietz MG (2020). P-REX1-Independent, Calcium-Dependent RAC1 Hyperactivation in Prostate Cancer. Cancers (Basel).

[34] Cao WX, Li T, Tang ZH (2018). MLKL mediates apoptosis via a mutual regulation with PERK/eIF2α pathway in response to reactive oxygen species generation. Apoptosis.

[35] Tkachenko A, Havranek O (2024). Erythronecroptosis: an overview of necroptosis or programmed necrosis in red blood cells. Mol Cell Biochem.

[36] Floride E, Föller M, Ritter M, Lang F (2008). Caffeine inhibits suicidal erythrocyte death. Cell Physiol Biochem.

[37] Schorn C, Frey B, Lauber K (2011). Sodium overload and water influx activate the NALP3 inflammasome. J Biol Chem.

[38] Okada Y, Maeno E, Shimizu T, Dezaki K, Wang J, Morishima S (2001). Receptor-mediated control of regulatory volume decrease (RVD) and apoptotic volume decrease (AVD). J Physiol.

[39] Al Mamun Bhuyan A, Nguyen MT, Bissinger R, Götz F, Lang F (2017). Lipopeptide-Induced Suicidal Erythrocyte Death Correlates with the Degree of Acylation. Cell Physiol Biochem.

[40] Mischitelli M, Jemaà M, Almasry M, Faggio C, Lang F (2016). Triggering of Erythrocyte Cell Membrane Scrambling by Emodin. Cell Physiol Biochem.

[41] Banerjee A, Chattopadhyay A, Pal PK, Bandyopadhyay D (2020). Melatonin is a potential therapeutic molecule for oxidative stress induced red blood cell (RBC) injury : A review. Melatonin Research.

[42] Repsold L, Joubert AM (2018). Eryptosis: An Erythrocyte’s Suicidal Type of Cell Death. Biomed Res Int.

[43] Kot Y, Prokopiuk V, Klochkov V (2025). Mn3O4 Nanocrystal-Induced Eryptosis Features Ca^2+^ Overload, ROS and RNS Accumulation, Calpain Activation, Recruitment of Caspases, and Changes in the Lipid Order of Cell Membranes. Int J Mol Sci.

[44] Manrique-Moreno M, Londoño-Londoño J, Jemioła-Rzemińska M (2014). Structural effects of the Solanum steroids solasodine, diosgenin and solanine on human erythrocytes and molecular models of eukaryotic membranes. Biochim Biophys Acta.

[45] Lang KS, Myssina S, Lang PA (2004). Inhibition of erythrocyte phosphatidylserine exposure by urea and Cl. Am J Physiol Renal Physiol.

